# Influence of cryoablation versus operation on circulating lymphocyte subsets in patients with early-stage renal cell carcinoma

**DOI:** 10.1186/s12885-024-12596-w

**Published:** 2024-07-10

**Authors:** Johanna Waidhauser, Anna-Katharina Gantner, Paola Schifano, Katharina Rippel, Stefan Schiele, Tim Tobias Arndt, Gernot Müller, Julie Steinestel, Andreas Rank, Thomas Kröncke

**Affiliations:** 1 Department of Hematology and Oncology, University Medical Center Augsburg, Stenglinstr.2, 86156 Augsburg, Germany; 2 Department of Urology, University Medical Center Augsburg, Augsburg, Germany; 3 Department of Diagnostic and Interventional Radiology, University Medical Center Augsburg, Augsburg, Germany; 4 Institute of Mathematics, Faculty of Mathematics and Natural Sciences, University of Augsburg, Augsburg, Germany; 5 General Pathology and Molecular Diagnostics, Medical Faculty, University of Augsburg, Augsburg, Germany

**Keywords:** Renal cell carcinoma, Cryoablation, Surgery, Flow cytometry, Lymphocytes

## Abstract

Immune response is known to play an important role in local tumor control especially in renal cell carcinoma (RCC), which is considered highly immunogenic. For localized tumors, operative resection or local ablative procedures such as cryoablation are common therapeutical options. For thermal ablative procedures such as cryoablation, additional immunological anti-tumor effects have been described.

The purpose of this prospective study was to determine changes in peripheral blood circulating lymphocytes and various of their subsets in RCC patients treated with cryoablation or surgery in a longitudinal approach using extensive flow cytometry. Additionally, lymphocytes of RCC patients were compared to a healthy control group.

We included 25 patients with RCC. Eight underwent cryoablation and 17 underwent surgery. Univariate and multivariable analysis revealed significantly lower values of B cells, CD4 and CD8 T cells, and various of their subsets in the treatment groups versus the healthy control group. Comparing the two different therapeutical approaches, a significant decline of various lymphocyte subsets with a consecutive normalization after three months was seen for the surgery group, whereas cryoablation led to increased values of CD69 + CD4 + and CD69 + CD8 + cell counts as well as memory CD8 + cells after three months.

Treatment-naïve RCC patients showed lower peripheral blood lymphocyte counts compared to healthy controls. The post-treatment course revealed different developments of lymphocytes in the surgery versus cryoablation group, and only cryoablation seems to induce a sustained immunological response after three months.

**Supplementary Information:**

The online version contains supplementary material available at 10.1186/s12885-024-12596-w.

## Introduction

Immune response is known to play an important role in local tumor control of renal cell carcinoma (RCC), and RCC is considered as one of the most immunogenic tumors, showing the highest rates of T cell infiltration compared to other kinds of cancer [1]. Although higher rates of infiltrating T cells are associated with poorer prognosis in general [2], differentiation between T-cell subgroups is needed. Whereas regulatory T cells and Th2 cells are associated with a poorer prognosis, higher rates of cytotoxic T cells are associated with better prognosis [1, 3, 4]. This high immunogenicity is exploited by immune-mediated therapeutic approaches in the treatment of advanced RCC. Starting with Interleukin 2 and Interferon α as non-specific immunotherapy, checkpoint inhibitors are now the main players in the first line treatment of metastasized clear cell renal cancer [5]. Nephrectomy or, if feasible regarding tumor size and localization, partial nephrectomy is considered the first line therapy for localized tumors [6]. For patients not eligible for surgery due to age or comorbidities and tumors sized < 3 cm, local ablative procedures are gaining importance and are a promising approach [6–8]. For thermal ablative procedures such as radiofrequency ablation (RFA) or cryoablation, immunological anti-tumor effects have been described in animal models and in humans [9, 10]; however, clinical significance remains controversial.

Whether the described high immunogenicity of renal cell carcinomas is also reflected by alterations in circulating lymphocyte subsets and whether a potential influence of surgery or cryoablation can be seen in peripheral blood lymphocytes has not sufficiently been studied so far. In our prospective study, we investigate the potential differences in peripheral blood B cells, T cells, and NK cells as well as various of their subsets between patients with localized RCC and a healthy control group and address the question of whether the therapeutical method (cryoablation or surgery) has an impact on these cells in a longitudinal, hypothesis-generating approach.

## Methods

### Study design and patient selection

We included patients with newly diagnosed localized and histologically confirmed RCC who were either scheduled for surgery or for cryoablation according to the recommendation of the local tumor board between June 2020 and March 2021. Patients with chronic infections (especially hepatitis or HIV), a known history of autoimmune disorder, or inherent or acquired immunodeficiency were excluded. Additionally, a control group of healthy volunteers, mostly blood donors, were recruited. Written informed consent was obtained from patients and healthy controls. The study was approved by the medical ethical committee of Ludwig Maximilians University Munich (reference number N20-001).

### Cryoablation and operation

Cryoablation was performed after diagnostic imaging by multi-detector spiral computed tomography (Siemens Somatom Definition AS, Forchheim, Germany) for treatment planning. Percutaneous CT-guided placement of up to five 17G cryoprobes (IceForce®, IceRod®, or IceSphere®; Boston Scientific, St Paul, MN, USA) into the tumor with an interprobe distance of less than 1 cm ensuring complete coverage of the tumor including a > 5 mm safety margin was performed. Carbon dioxide or hydrodissection were utilized when appropriate to protect nearby structures. Cryoablation was performed with an argon-based cryoablation system (Visual Ice; Boston Scientific, St Paul, MN, USA) applying two freeze–thaw cycles as follows: 10 min of freezing with 100% freeze intensity (uninterrupted argon flow) with consecutive passive (8 min) and active (2 min) thawing while CT scans to assess expansion of the ice-ball were obtained at regular 4-min intervals. An effective core temperature of minus 40 degrees Celsius, variable in size depending on the type of cryoprobe, can be expected according to the manufacturer. After the procedure, patients were taken to a recovery area for monitoring and were subsequently sent to the ward for overnight surveillance and discharged the next day according to the local discharge policy.

Operative tumor removal was performed using complete or, if feasible, partial nephrectomy according to the urologist’s assessment.

### Analysis of lymphocytes and subsets

EDTA blood samples were taken before (V0), one day after (V1), one week after (V2), and three months after (V3) operative tumor resection or cryoablation. Flow cytometry was performed within 24 h in our local laboratory at University Medical Center Augsburg using FC500 (Beckman Coulter, Brea, California, USA) according to previously published studies by our research group [11]. Cell staining was done using commercial antibodies purchased from Beckman Coulter (Brea, California, USA) and Biolegend (San Diego, California, USA). Initial results for lymphocyte subsets were given as percentages. Absolute values were calculated using absolute leucocyte counts measured with Stem-Count (Stem-Kit, Beckman Coulter).

Detailed information regarding flow cytometric identification of lymphocyte subsets, antibodies, and gating strategy is provided in Supplement Figure S1 [12], Table S1, and Table S2.

### Charlson comorbidity Index

For all patients, we calculated the Charlson Comorbidity Index according to prior publications to quantify relevant comorbidities [13]. Healthy controls were rated with zero points, as pre-existing medical conditions would have excluded them from blood donation according to the national guidelines of blood donation.

### Statistical analysis

The results of the descriptive analysis are given as median values and interquartile ranges. To detect differences between patients before treatment and the control group, as well as female and male study populations, we performed univariate analysis using the Mann–Whitney U test. We analyzed age-dependent alterations of lymphocyte subsets after logarithmic transformation and additionally performed a multivariable linear regression analysis on log-transformed cell counts including age, gender, and the presence of RCC. The results of the multivariable analysis were given as multiplicative factors (coefficient B). To detect changes in cell counts from V0 to the other timepoints (V1–V3), paired t-tests on the log-transformed cell counts were performed. *P* values < 0.05 were considered statistically significant. Data were analyzed with SPSS for Windows (IBM SPSS Statistics 24, Armonk, New York, USA) and R 4.0.2 (R Foundation for Statistical Computing, Vienna, Austria).

## Results

### Population characteristics

We recruited a total of 25 consecutive RCC patients according to the inclusion criteria, of which 8 underwent cryoablation and 17 underwent conventional surgery, and additionally analyzed the blood of 50 healthy individuals comprising the control group. The median age of the RCC patients was 69 (range 40–93) years versus 43 (range 18–81) years in the control group (*p* < 0.001). There was also a significant age difference (*p* = 0.019) between the cryoablation group (median 77, range 66–87 years) and the surgery group (median 63, range 40–93 years). Further information on the demographic and disease characteristics is displayed in Table 1. T stage was determined based on prior imaging for the cryoablation and on final pathology for the surgery group and varied between T1a and T3a.

**Table 1:** Demographic and disease characteristics of patients and healthy control group

**Variables**	**Patients** (*n* = 25)	**Control group** (*n* = 50)	***p*-value** (Patients vs. Control)	**Cryoablation** (*n* = 8)	**Operation** (*n* = 17)	***p*-value** (Cryoablation vs. Operation)
Age; *median (range)*	69 (40–93)	43 (18–81)	.000	77 (66–87)	63 (40–93)	.019
Sex						
male;* n (%)*	18 (72)	33 (66)	n.s	5 (63)	13 (76)	n.s
female; *n (%)*	7 (28)	17 (34)		3 (37)	4 (24)	
Histology						
Clear cell	9			3	6	
Chromophobe	9			3	6	
Papillary	7			2	5	
T Stage						
T1a	14			7	8	
T1b	3			1	2	
T2a	3				3	
T3a	5			0	4	
Charlson Comorbidity Index; *mean (range)*	3,6 (2–9)			4.5 (2–9)	3.2 (2–6)	n.s
Operative Procedure						
Nephrectomy					6	
Partial Nephrectomy					11	

### Univariate analysis of lymphocyte subsets before therapy

**Fig. 1:**
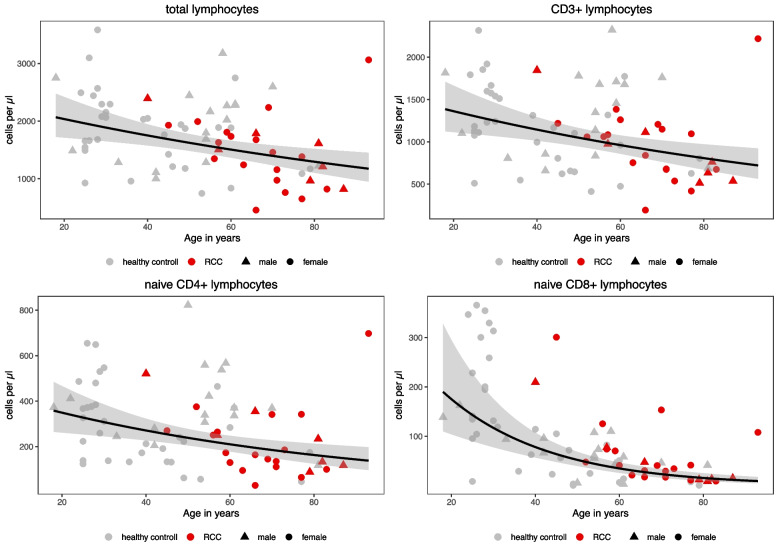
Age-dependent distribution of cell counts and median decrease over lifetime. Black line = smoothed means of log transformed data, 95% confidence interval in gray

Univariate analysis revealed significantly lower lymphocyte counts for treatment-naïve RCC patients compared to healthy controls for total lymphocytes (median: 1459/µl vs. 1878/µl; *p* = 0.010); total B cells (110/µl vs. 206/µl; *p* = 0.001) and all of their subsets; total T cells (974/µl vs. 1175/µl; *p* = 0.018); CD4 cells (589/µl vs. 765/µl; *p* = 0.042) and their subsets of naïve CD4, effector memory CD4, EMRA CD4, CD69 + CD4, Th1, and regulatory (CD25 +) CD4 cells; CD8 cells (158/µl vs. 292/µl; *p* = 0.001) and their subsets of memory CD8, naïve CD8, effector memory CD8, and early, intermediate, late, and exhausted CD8 cells; as well as CD69 + CD8 + cells (see Table 2). Sex had a significant influence on naïve CD4 (339/µl vs. 223/µl; *p* = 0.049) and CD69 + CD4 cells (13/µl vs. 8/µl; *p* = 0.009), Th2 cells (71/µl vs. 45/µl; *p* = 0.020), and the CD4/CD8 ratio (3.6 vs. 2.1; *p* < 0.001), with higher values for women. Age had a significant influence with lower values of total lymphocytes; B cells and their subsets of transitory, naïve, and memory B cells; CD3 + cells; and CD4 + cells and CD8 + cells and various of their subsets. The strongest effect was seen for naïve CD4 + and naïve CD8 + cells (Fig. 1).

**Table 2:** Lymphocyte counts in healthy controls and patients at different time points (V0 = before procedure, V1 = after one day, V2 = after one week, V3 = after three months). Cell counts are given as median value/ µl (interquartile range). *P*-values are given for univariate analysis between total group of cancer patients before treatment and healthy controls

	**NCC patients (*n* = 25)** **V0**	**NCC patients (*n* = 25)** **V1**	**NCC patients (*n* = 25)** **V2**	**NCC patients (*n* = 25)** **V3**	**Control group** **(*n* = 50)**	***p*-value NCC patients V0** **vs. Control**
Total lymphocytes	1459 (970–1796)	1100 (677–1386)	1021 (820–1485)	1345 (1095–1803)	1878 (1282–2293)	.010
B lymphocytes	110 (53–197)	63 (46–205)	89 (38–162)	97 (46–150)	206 (146–276)	.001
transitory B	2 (1–8)	2 (1–11)	2 (1–6)	6 (1–13)	10 (5–18)	< .001
naïve B	58 (30–142)	50 (27–137)	51 (21–104)	64 (21–113)	126 (90–172)	.002
memory B	4 (2–10)	3 (1–7)	2 (2–4)	3 (2–8)	7 (5–13)	.009
class-switched B	12 (8–20)	9 (5–22)	9 (5–19)	12 (7–17)	24 (14–43)	.003
CD3 cells	974 (654–1178)	673 (443–985)	724 (515–996)	960 (667–1181)	1175 (806–1678)	.018
CD4_CD8	2 (1–7)	3 (1–6)	3 (1–5)	3 (2–7)	3 (2–7)	.719
CD4 cells	589 (438–777)	488 (296–640)	516 (375–653)	597 (475–812)	765 (525–1017)	.042
memory CD4	406 (311–460)	349 (153–416)	317 (226–428)	420 (348–504)	405 (309–590)	.323
naïve CD4	173 (114–306)	129 (71–237)	167 (97–262)	150 (112–301)	311 (173–417)	.013
CM CD4	292 (219–345)	203 (126–311)	233 (139–320)	313 (189–386)	269 (157–403)	.511
EM CD4	103 (77–135)	81 (39–113)	71 (50–124)	107 (83–173)	143 (93–238)	.022
EMRA CD4	1 (1–7)	1 (0–5)	1 (0–7)	2 (0–6)	4 (1–32)	.013
HLADR CD4	51 (31–76)	40 (23–53)	40 (23–61)	61 (38–80)	39 (27–60)	.134
CD69 CD4	8 (6–9)	7 (4–16)	10 (6–16)	8 (6–11)	12 (7–19)	.038
Th1	12 (6–31)	9 (4–19)	8 (5–19)	13 (7–25)	27 (14–58)	.001
Th2	54 (41–90)	43 (24–74)	47 (27–67)	58 (38–97)	45 (30–67)	.136
Th17	67 (36–86)	50 (25–75)	50 (32–66)	62 (46–102)	60 (35–82)	.727
IL2 (CD25 +) CD4	8 (6–13)	6 (4–11)	6 (4–10)	8 (5–11)	17 (8–21)	< .001
CD8 cells	158 (109–322)	138 (88–245)	162 (98–267)	197 (103–341)	292 (203–493)	.001
memory CD8	67 (40–113)	49 (31–112)	60 (29–90)	75 (53–112)	118 (63–165)	.023
naïve CD8	40 (16–75)	34 (15–61)	32 (13–57)	37 (16–72)	82 (35–140)	.045
CM CD8	23 (15–46)	18 (13–31)	20 (10–36)	25 (18–49)	29 (13–55)	.619
EM CD8	47 (32–84)	33 (22–81)	38 (21–75)	55 (40–86)	94 (55–144)	.004
EMRA CD8	40 (18–69)	25 (10–53)	36 (10–53)	49 (23–75)	51 (21–129)	.199
early CD8	105 (56–231)	102 (45–159)	102 (43–143)	98 (65–214)	180 (131–295)	.002
intermediate CD8	12 (5–22)	12 (3–22)	13 (3–24)	19 (6–26)	21 (12–34)	.012
late CD8	24 (14–79)	23 (7–49)	29 (8–73)	37 (13–68)	51 (26–124)	.045
exhausted CD8	42 (23–75)	46 (19–64)	49 (20–78)	68 (30–94)	86 (54–136)	.001
terminal effector CD8	22 (6–37)	11 (6–30)	14 (5–38)	22 (11–37)	24 (12–95)	.163
HLADR CD8	29 (10–66)	23 (9–51)	27 (9–56)	27 (14–78)	31 (17–61)	.694
CD69 CD8	12 (8–23)	15 (6–25)	23 (9–31)	18 (10–42)	32 (17–89)	< .001
IL2 (CD25 +) CD8	0 (0–1)	0 (0–1)	0 (0–1)	1 (0–1)	1 (0–1)	.093
NKT	19 (12–60)	17 (6–35)	16 (9–58)	33 (15–63)	45 (19–81)	.068
NK	177 (155–295)	141 (92–221)	131 (54–240)	187 (119–309)	226 (139–301)	.770
D56dimCD16bright	17 (9–25)	10 (5–22)	10 (5–18)	18 (10–28)	15 (10–20)	.678
CD56brightCD16bright	149 (120–254)	126 (59–187)	111 (42–195)	161 (98–279)	193 (111–274)	.621
CD56brightCD16dim	11 (7–19)	7 (3–14)	5 (3–10)	14 (8–20)	15 (11–19)	.147
CD4/CD8 Ratio	4 (2–5)	3 (2–5)	3 (2–6)	3 (2–4)	2 (2–3)	.024

Due to these significant differences, the factors RCC, gender, and age were included in a multivariable analysis. As the CCI did not show significant differences between the control group and patients, it was not included in the multivariable analysis.

### Multivariable analysis of lymphocytes before therapy

**Fig. 2:**
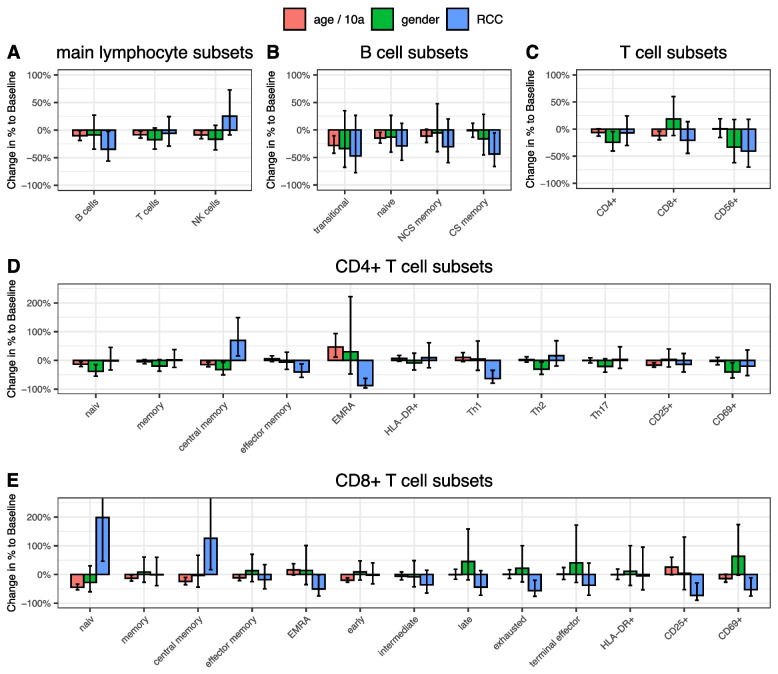
Impact of age, gender, and renal cell carcinoma on lymphocyte subsets as calculated in multivariable analysis. **A** Main lymphocyte subsets, **B** B cell subsets, **C** T cell subsets, **D** CD4 + T cell subsets, **E** CD8 + T cell subsets

Multivariable analysis before the therapeutic procedure revealed an influence of age, with lower values of absolute lymphocyte count; B cells, especially naïve and transitory B cells; naïve and central memory CD4 + cells; total CD8 + cells and their subgroups of memory, naïve, central memory and early CD8 + cells; and NK cells and their subgroups of CD56 + CD16 + and CD56brightCD16dim cells. Women had higher quantities of CD4 + cells and their subgroups of naïve, central memory, CD69 + , and Th2 cells as well as CD56brightCD16dim NK cells. Regarding the influence of the RCC on lymphocyte count, significantly lower values could be seen for total and class-switch B cells, and effector memory and EMRA CD4 + cells, as well as Th1 cells and for EMRA, exhausted, CD69 + , and regulatory CD8 + cells. Higher values in RCC patients were only seen for naïve CD8 + cells and central memory CD4 + and CD8 + cells (Fig. 2*, ****Table S3***).

### Longitudinal comparison between surgery and cryoablation

**Fig. 3:**
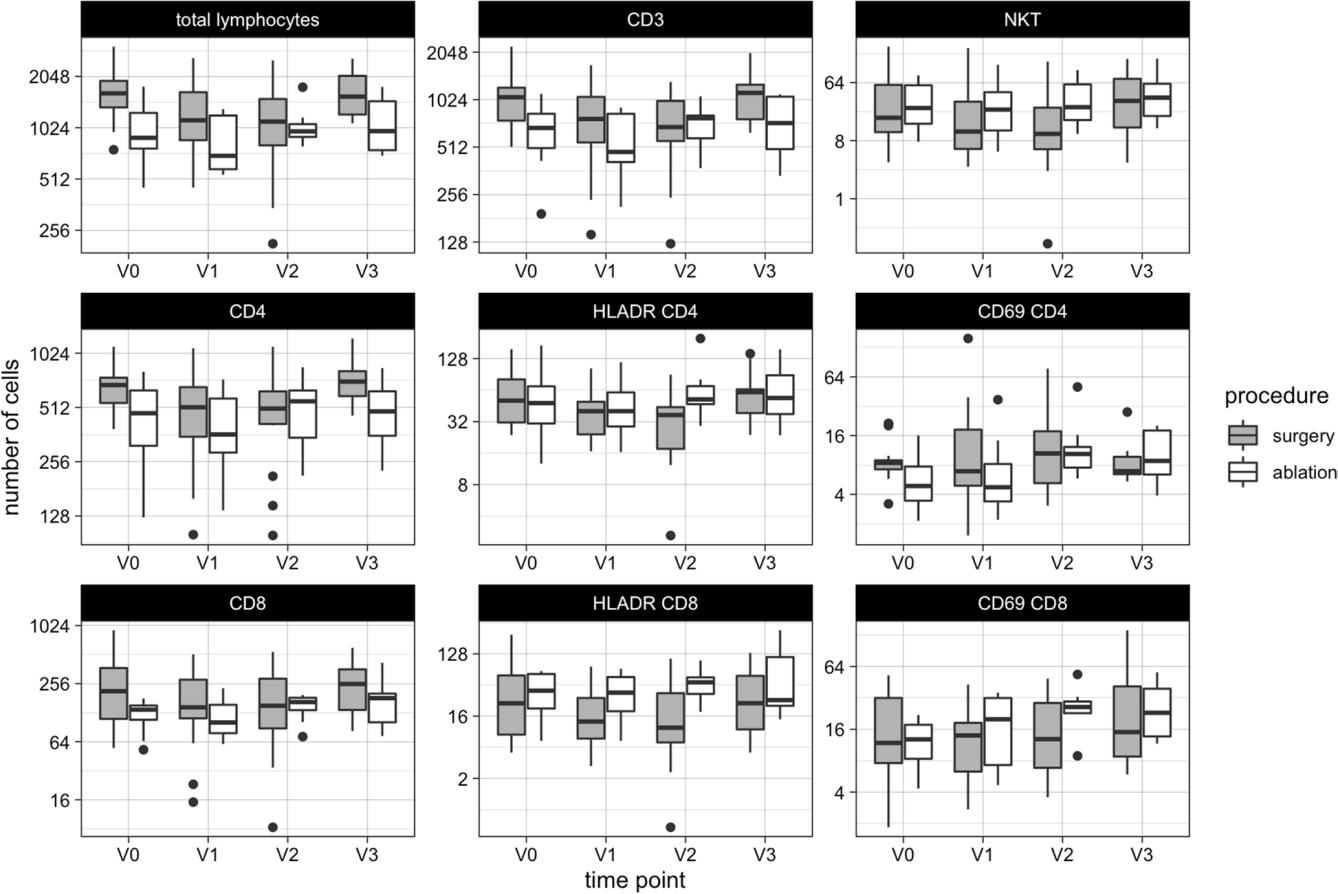
Longitudinal development of selected lymphocyte subsets (cells/ µl) in surgery and ablation groups. Box plots display median values and interquartile ranges

Comparing the lymphocyte values before the therapeutical procedure with those one day after surgery revealed a significant decrease in total lymphocytes (1630/ µl vs. 1132/µl; *p* = 0.018), total T cells (1061/µl vs. 771/µl; *p* = 0.011), CD4 + cells (682/µl vs. 513/µl; *p* = 0.020) and their subgroups of memory (406/µl vs. 356/µl; *p* = 0.031), central memory (296/µl vs. 244/µl; *p* = 0.022) and naïve CD4 + cells (234/µl vs. 130/µl; *p* = 0.004), as well as Th1 (14/µl vs. 10/µl; *p* = 0.016) and Th2 cells (64/µl vs. 48/µl; *p* = 0.034), naïve CD8 + (48/µl vs. 40/µl; *p* = 0.039), EMRA CD8 + (40/µl vs. 23/µl; *p* = 0.022), late CD8 + (21/µl vs. 20/µl; *p* = 0.044), terminal effector CD8 + (22/µl vs. 11/µl; *p* = 0.005), NKT cells (18/µl vs. 11/µl; *p* = 0.015), NK cells (183/µl vs. 129/µl; *p* = 0.001) and all of their subsets. Regarding B cells, only memory B cells showed a significant decline at this time point after surgery (15/µl vs. 12/µl; *p* = 0.017). After one week (V2), additional changes could be seen for total B cells (183/µl vs. 136/µl; *p* = 0.003) and naïve (86/µl vs. 59/µl; *p* < 0.001), transitory (4/µl vs. 3/µl; *p* = 0.004), and class-switch B cells (15/µl vs. 13/µl; *p* = 0.013), which all showed lower values at this time point compared to the preoperative values in the surgery group. For T cells, additional declines could be seen for effector memory CD4 + (115/µl vs. 61/µl; *p* = 0.011), HLADR CD4 + (51/µl vs. 37/µl; *p* = 0.004), Th17 (69/µl vs. 54/µl; *p* = 0.007), regulatory (CD25 +) CD4 + cells (9/µl vs. 6/µl; *p* = 0.035), CD8 + cells (214/µl vs. 151/µl; *p* = 0.006) and their subsets of memory (71/µl vs. 53/µl; *p* = 0.007), central memory (32/µl vs. 20/µl; *p* = 0.007), effector memory (47/µl vs. 27/µl; *p* = 0.009), early (136/µl vs. 104/µl; *p* = 0.010), and intermediate CD8 + cells (9/µl vs. 5/µl; *p* = 0.030), as well as HLADR CD8 + cells (25/µl vs. 11/µl; *p* = 0.005) one week after surgery. Almost all subsets that showed significantly lower values the day after surgery remained reduced after one week with mostly even stronger declines. Three months after surgery, none of the reported declines persisted. The only significant change that could be seen in the surgery group at this time point was an increase of regulatory (CD25high) CD8 + cells (0/µl vs. 1/µl; *p* = 0.030) (Fig. 3***, ***Table 3).

**Table 3:** Lymphocyte counts in different study groups (surgery/ablation) at different time points (V0 = before procedure, V1 = after one day, V2 = after one week, V3 = after three months). Cell counts are given as median value/µl (interquartile range). *P*-values are given for longitudinal comparisons

	**Ablation (*n*** **= 8)** **V0**	**Ablation (*n*** **= 8)** **V1**	**Ablation (*n*** **= 8)** **V2**	**Ablation (*n*** **= 8)** **V3**	***p*-value V0 vs.**		
					**V1**	**V2**	**V3**
**Total lymphocytes**	897 (692 - 1340)	701 (569 - 1212)	975 (837 - 1142)	1003 (723 - 1529)	0.3780	0.2821	0.3076
**B lymphocytes**	54 (29 - 87)	47 (24 - 71)	41 (21 - 93)	63 (25 - 93)	0.1904	0.1823	0.5425
** transitory B**	1 (0 - 3)	1 (0 - 2)	1 (0 - 4)	2 (0 - 6)	0.1492	0.0029	0.6878
** naiv B**	34 (12 - 58)	24 (11 - 56)	23 (8 - 55)	40 (7 - 66)	0.1995	0.1735	0.3628
** memory B**	3 (1 - 4)	2 (1 - 4)	2 (1 - 3)	2 (1 - 3)	0.0204	0.3223	0.4772
** class switched B**	7 (6 - 10)	6 (3 - 9)	5 (4 - 8)	7 (5 - 10)	0.2578	0.3547	0.6768
**CD3 cells**	677 (448 - 1011)	477 (382 - 847)	781 (516 - 863)	733 (439 - 1076)	0.3752	0.1921	0.3196
**CD4_CD8**	2 (1 - 5)	3 (1 - 5)	4 (1 - 6)	3 (0 - 8)	0.3737	0.5634	0.5665
**CD4 cells**	479 (293 - 751)	363 (250 - 588)	556 (319 - 653)	489 (310 - 754)	0.5008	0.1401	0.3195
** memory CD4**	367 (203 - 453)	300 (148 - 385)	365 (233 - 507)	358 (245 - 462)	0.5025	0.0832	0.2563
** naiv CD4**	114 (73 - 290)	88 (60 - 214)	121 (71 - 246)	108 (65 - 289)	0.4973	0.5684	0.6654
** CM CD4**	242 (133 - 350)	198 (117 - 262)	247 (143 - 333)	224 (177 - 355)	0.5321	0.2742	0.2243
** EM CD4**	80 (49 - 139)	94 (43 - 116)	105 (66 - 140)	87 (64 - 157)	0.5310	0.0266	0.3583
** EMRA CD4**	1 (0 - 7)	1 (0 - 5)	4 (1 - 7)	1 (0 - 4)	0.9074	0.1125	0.4306
** HLADR CD4**	49 (30 - 70)	40 (21 - 82)	52 (45 - 77)	55 (36 - 102)	0.5497	0.0446	0.1449
** CD69 CD4**	5 (3 - 11)	5 (3 - 12)	10 (7 - 15)	9 (5 - 18)	0.5215	0.0174	0.0007
** Th1**	7 (7 - 29)	7 (4 - 39)	9 (6 - 37)	8 (6 - 25)	0.7297	0.6617	0.5357
** Th2**	38 (24 - 58)	36 (17 - 46)	43 (25 - 67)	40 (29 - 54)	0.5166	0.9841	0.2318
** Th17**	45 (27 - 66)	52 (21 - 64)	43 (32 - 75)	50 (34 - 66)	0.6029	0.1339	0.0747
** IL2 CD4**	6 (3 - 8)	4 (3 - 5)	6 (4 - 8)	6 (5 - 7)	0.0604	0.6813	0.8596
**CD8 cells**	138 (81 - 156)	101 (77 - 196)	165 (114 - 189)	182 (101 - 210)	0.7672	0.1453	0.0797
** memory CD8**	58 (31 - 77)	47 (41 - 60)	86 (44 - 93)	66 (53 - 93)	0.8104	0.1632	0.0428
** naiv CD8**	16 (12 - 38)	16 (11 - 33)	23 (10 - 32)	16 (14 - 40)	0.9114	0.8758	0.5349
** CM CD8**	18 (10 - 23)	16 (14 - 22)	21 (13 - 28)	23 (15 - 28)	0.7437	0.4504	0.0131
** EM CD8**	46 (22 - 58)	35 (26 - 49)	62 (33 - 78)	55 (42 - 75)	0.9144	0.0853	0.0621
** EMRA CD8**	41 (13 - 50)	28 (18 - 50)	43 (23 - 58)	47 (17 - 78)	0.8226	0.0663	0.1154
** early CD8**	55 (35 - 98)	48 (37 - 90)	76 (43 - 128)	70 (46 - 103)	0.6575	0.2302	0.2312
** intermediate CD8**	13 (10 - 18)	15 (10 - 22)	17 (14 - 24)	22 (13 - 26)	0.6512	0.0145	0.1667
** late CD8**	37 (11 - 68)	30 (14 - 56)	34 (13 - 82)	45 (9 - 114)	0.9589	0.3994	0.4449
** exhausted CD8**	48 (17 - 80)	50 (30 - 65)	64 (51 - 85)	55 (23 - 93)	0.3072	0.0529	0.2618
** terminal effector CD8**	19 (6 - 31)	13 (8 - 18)	20 (10 - 38)	22 (11 - 58)	0.7161	0.8076	0.1807
** HLADR CD8**	37 (15 - 67)	36 (14 - 62)	50 (28 - 62)	27 (19 - 127)	0.6550	0.1980	0.1166
** CD69 CD8**	13 (8 - 19)	20 (6 - 32)	26 (23 - 32)	23 (12 - 42)	0.1474	0.0121	0.0034
** IL2 CD8**	1 (0 - 1)	1 (0 - 1)	1 (0 - 1)	0 (0 - 1)	0.2516	0.4227	0.6034
**NKT**	26 (13 - 60)	25 (9 - 71)	28 (15 - 65)	41 (19 - 63)	0.5705	0.3442	0.0255
**NK**	156 (109 - 303)	152 (109 - 237)	181 (131 - 264)	288 (95 - 323)	0.2856	0.4503	0.3885
** D56dimCD16bright**	19 (9 - 25)	13 (7 - 34)	16 (7 - 21)	19 (13 - 28)	0.8912	0.3983	0.6392
** CD56brightCD16bright**	135 (80 - 269)	129 (73 - 181)	154 (101 - 244)	247 (72 - 303)	0.2311	0.2938	0.3636
** CD56brightCD16dim**	13 (7 - 23)	8 (6 - 21)	10 (8 - 23)	11 (6 - 24)	0.0203	0.8794	0.2213
**CD4/CD8 Ratio**	4 (2 - 5)	3 (2 - 4)	4 (2 - 4)	4 (2 - 4)	0.2675	0.2455	0.0941
	**Operation (*n*** **= 17)** **V0**	**Operation (*n*** **= 17)** **V1**	**Operation (*n*** **= 16)** **V2**	**Operation (*n*** **= 12)** **V3**	***p*-value V0 vs.**		
					**V1**	**V2**	**V3**
**Total lymphocytes**	1630 (1295 - 1960)	1132 (795 - 1688)	1113 (697 - 1528)	1560 (1181 - 2203)	0.0182	0.0029	0.9179
**B lymphocytes**	183 (82 - 251)	191 (55 - 272)	136 (62 - 203)	134 (97 - 254)	0.3541	0.0029	0.6055
** transitory B**	4 (1 - 12)	3 (1 - 15)	3 (1 - 7)	8 (3 - 15)	0.4175	0.0044	0.8161
** naiv B**	86 (50 - 163)	76 (34 - 205)	59 (36 - 117)	82 (40 - 176)	0.6622	0.0003	0.3679
** memory B**	6 (2 - 12)	6 (1 - 10)	3 (2 - 5)	7 (2 - 9)	0.0713	0.0021	0.4201
** class switched B**	15 (11 - 38)	12 (6 - 29)	13 (8 - 23)	16 (12 - 33)	0,0167	0.0133	0.6390
**CD3 cells**	1061 (714 - 1240)	771 (503 - 1089)	686 (515 - 1019)	1131 (761 - 1404)	0.0105	0.0038	0.5236
**CD4_CD8**	3 (1 - 8)	3 (1 - 6)	3 (1 - 5)	4 (2 - 7)	0.8023	0.6059	0.7711
**CD4 cells**	682 (525 - 819)	513 (317 - 723)	505 (410 - 690)	715 (591 - 828)	0.0200	0.0100	0.5520
** memory CD4**	406 (350 - 505)	356 (153 - 430)	308 (166 - 401)	441 (398 - 533)	0.0307	0.0058	0.5760
** naiv CD4**	234 (140 - 306)	130 (103 - 256)	181 (105 - 311)	195 (140 - 301)	0.0040	0.0611	0.9731
** CM CD4**	296 (250 - 345)	244 (126 - 318)	224 (121 - 300)	348 (280 - 390)	0.0221	0.0074	0.8763
** EM CD4**	115 (85 - 135)	78 (35 - 138)	61 (41 - 123)	131 (98 - 178)	0,.0621	0.0110	0.2353
** EMRA CD4**	2 (1 - 7)	1 (0 - 5)	1 (0 - 14)	2 (1 - 14)	0.3245	0.2630	0.5521
** HLADR CD4**	51 (31 - 85)	40 (23 - 51)	37 (18 - 52)	61 (38 - 68)	0.0522	0.0042	0.3859
** CD69 CD4**	8 (7 - 9)	7 (4 - 19)	11 (5 - 22)	7 (6 - 10)	0.8818	0.4579	0.9878
** Th1**	14 (6 - 31)	10 (4 - 19)	6 (4 - 19)	14 (10 - 25)	0.0159	0.0324	0.2352
** Th2**	64 (43 - 95)	48 (24 - 75)	47 (27 - 70)	76 (54 - 112)	0.0337	0.0034	0.4007
** Th17**	69 (43 - 88)	49 (27 - 77)	54 (23 - 66)	76 (55 - 107)	0.0792	0.0065	0.1451
** IL2 CD4**	9 (7 - 14)	9 (5 - 13)	6 (4 - 13)	10 (7 - 13)	0.3715	0.0353	0.4347
**CD8 cells**	214 (109 - 420)	145 (107 - 309)	151 (74 - 290)	259 (117 - 386)	0.0620	0.0059	0.2683
** memory CD8**	71 (40 - 161)	58 (28 - 131)	53 (24 - 81)	83 (54 - 163)	0.0620	0.0068	0.4400
** naiv CD8**	48 (26 - 116)	40 (19 - 86)	45 (15 - 90)	61 (29 - 102)	0.0387	0.0221	0.8138
** CM CD8**	32 (15 - 53)	24 (11 - 54)	20 (10 - 39)	28 (19 - 52)	0.1248	0.0069	0.7912
** EM CD8**	47 (37 - 119)	31 (15 - 105)	27 (15 - 64)	64 (36 - 125)	0.0620	0.0091	0.4670
** EMRA CD8**	40 (18 - 74)	23 (8 - 55)	21 (9 - 52)	49 (26 - 62)	0.0223	0.0187	0.1146
** early CD8**	136 (73 - 256)	120 (49 - 178)	104 (38 - 170)	149 (85 - 260)	0.0788	0.0096	0.4078
** intermediate CD8**	9 (4 - 28)	7 (2 - 23)	5 (2 - 23)	15 (4 - 33)	0.0538	0.0302	0.5301
** late CD8**	21 (14 - 91)	20 (6 - 49)	20 (7 - 73)	34 (17 - 58)	0.0435	0.0339	0.1080
** exhausted CD8**	42 (28 - 75)	31 (18 - 77)	38 (15 - 53)	70 (30 - 111)	0.1610	0.0507	0.1058
** terminal effector CD8**	22 (6 - 51)	11 (3 - 37)	9 (5 - 40)	22 (11 - 37)	0.0050	0.0122	0.6109
** HLADR CD8**	25 (8 - 69)	13 (7 - 35)	11 (6 - 37)	25 (10 - 70)	0.0527	0.0046	0.3817
** CD69 CD8**	12 (8 - 33)	14 (6 - 21)	13 (6 - 30)	15 (8 - 57)	0.3744	0.8077	0.1879
** IL2 CD8**	0 (0 - 1)	0 (0 - 1)	0 (0 - 1)	1 (0 - 1)	0.9939	0.7901	0.0297
**NKT**	18 (8 - 70)	11 (6 - 33)	10 (6 - 48)	33 (12 - 103)	0.0152	0.0174	0.2054
**NK**	183 (162 - 295)	129 (86 - 221)	83 (51 - 149)	183 (132 - 246)	0.0010	0.0015	0.2134
** D56dimCD16bright**	15 (9 - 26)	7 (4 - 20)	9 (5 - 18)	18 (8 - 30)	0.0081	0.0782	0.7013
** CD56brightCD16bright**	151 (129 - 254)	116 (52 - 201)	68 (34 - 135)	151 (112 - 191)	0.0009	0.0010	0.1808
** CD56brightCD16dim**	11 (8 - 17)	6 (2 - 14)	4 (3 - 7)	14 (8 - 20)	0.0068	0.0007	0.8576
**CD4/CD8 Ratio**	3 (2 - 6)	3 (2 - 5)	3 (2 - 6)	3 (2 - 5)	0.4180	0.1050	0.3195

The type of surgery (partial vs. total nephrectomy) did not lead to significant differences between lymphocyte subsets at any timepoint in univariate analysis.

In the ablation group, no significant changes could be observed one day after the procedure with the exception of reduced memory B cells (3/µl vs. 2/µl; *p* = 0.020) and CD56brightCD16dim NK cells (13/µl vs. 10/µl; *p* = 0.020). One week after the ablation, an increase in effector memory CD4 + cells (80/µl vs. 105/µl; *p* = 0.027), HLADR CD4 + cells (49/µl vs. 52/µl; *p* = 0.045), CD69 + CD4 + cells (5/µl vs. 10/µl; *p* = 0.017), intermediate CD8 + cells (13/µl vs. 17/µl; *p* = 0.015), and CD69 + CD8 + cells (13/µl vs. 26/µl; *p* = 0.012) could be seen. Three months after therapy, CD69 + CD4 + cells (5/µl vs. 9/µl; *p* < 0.001) and CD69 + CD8 + cells (13/µl cs. 23/µl; *p* = 0.003) showed a persisting increase. Additionally, an increase in memory CD8 + cells (58/µl vs. 66/µl; *p* = 0.043), central memory CD8 + cells (18/µl vs. 23/µl; *p* = 0.013), and NKT cells (26/µl vs. 41/µl; *p* = 0.026) was found (Fig. 3***, ***Table 3).

## Discussion

Peripheral blood total lymphocyte counts, B cells, CD4 + cells, CD8 + cells and various of their subsets showed lower values in RCC patients compared to healthy controls in univariate analysis. Due to the known effects of age and gender [14, 15] on lymphocyte counts, these factors were included in a multivariable analysis in addition to the presence of cancer. Naïve CD8 + cells and central memory CD4 + and CD8 + cells showed higher values in RCC patients. Central memory T cells play an important role in tumor antigen recognition and tumor control, which could be an explanation for their higher counts in RCC patients [16, 17]. In the majority of lymphocyte subsets, especially in activated and memory CD4 + cells as well as different types of activated and memory CD8 + cells, significantly lower values were observed in RCC patients independently of age or gender. A similar effect has been reported in patients with head and neck cancer [18], and in colorectal carcinoma patients compared to healthy controls [11], while a study by Wang et al. revealed lower values for some lymphocyte subsets and higher values for others [19]. The underlying mechanism of these lower lymphocyte counts in RCC patients could be some kind of immunosuppression caused by the cancer or attraction of lymphocytes to the tumor, with resulting lower peripheral blood lymphocytes. To answer this pathophysiological question is beyond the scope of this study, and further investigations comprising, for example, tumor-infiltrating lymphocytes are needed.

Regarding the longitudinal development of peripheral blood lymphocytes, a decline of total lymphocytes, total T cells, and several subsets of CD4 + and CD8 + cells could be seen already one day after surgery with a prolonged and in parts even stronger effect after one week. This decline resolved after three months. It is known that surgical procedures can induce alterations of peripheral blood lymphocytes, which has been shown for cytotoxic T cells in patients after resection of hepatocellular carcinoma [20], and for total T cells and CD4 + cells in patients with gastrointestinal cancer [21].

In contrast to patients who underwent surgery, cryoablation had no remarkable effect on circulating lymphocytes after one day. One week after the procedure, an increase in different types of CD4 + and CD8 + cells could be observed including mainly activated cells as CD69 + CD4 + cells, HLADR CD4 + cells, and CD69 + CD8 + cells. Higher values of CD69 + CD4 + and CD69 + CD8 cells persisted after three months. This effect might be caused by the differing therapeutical approach. The intention of cryoablation is the destruction of tumor tissue with a resulting release to the bloodstream and antigen presentation to lymphocytes [22], while surgery tends to be performed using a no-touch technique to avoid the circulation of tumor cells. Immune stimulation after cryoablation is dependent on tumor cell necrosis, while apoptosis seems to induce immunosuppression [23]. The effects of cryoablation-induced immune activation on long-term prognosis are controversial [22], and longer-lasting effects have been described for combination with other immunotherapeutic approaches [24, 25]. The increase in activated T cells three months after cryoablation in our study population could be evidence for a persisting immune activation in this group of patients in contrast to the patients who underwent surgery. HLA-DR positive cells in particular seem to be crucial for immunological tumor control, as HLA-DR loss is linked to tumor escape from immunosurveillance [26], and higher numbers of activated HLA-DR + CD4 + cells were associated with a better prognosis in B-NHL patients [27]. For breast cancer patients, a positive correlation of tumor-infiltrating and circulating HLA-DR positive cytotoxic T cells has been shown with lower rates of lymph node metastases and better response to chemotherapy in HLA-DR^hi^ patients [28]. However, some studies report adverse prognostic effects of elevated HLA-DR T cells in patients with solid tumors or hematological malignancies [29, 30]. CD69 + T cells display another type of activated cells for which an important role in anti-tumor immunity has been reported [31]. Whether elevated activated T cells have an impact on long-term prognosis can, however, not be answered by our study population.

The question whether surgical technique (partial vs. total nephrectomy) makes a difference cannot be finally answered. Comparing these two subgroups revealed no significant differences in lymphocyte subsets at the different time points in univariate analysis. Because of this and the small sample size no multivariate analysis including this parameter was performed.

Missing long time follow-up is one limitation of our study, as prognostic differences between surgery and ablation, possibly caused by the different development of lymphocyte counts, cannot be detected. Further, we are unable to state whether the cryoablation-induced effect of cellular immune system activation seen at three months was specifically directed against RCC. Another limitation is the relatively small number of patients in the ablation group. However, longitudinal comparison revealed significant changes of lymphocyte counts regardless of the low patient numbers.

In summary, our data show that lymphocyte counts of RCC patients are lower than those of healthy controls and that the two therapeutical approaches of surgery and cryoablation lead to different developments of circulating lymphocytes. Cryoablation induces a sustained activation of CD4 + and CD8 + T cells three months after the procedure. This observation may indicate a longer-term immunostimulatory effect of cryoablation, which could support immunological tumor control.

### Supplementary Information

Additional file 1.

## Data Availability

The datasets used and/or analysed during the current study are available from the corresponding author on reasonable request.
