# Supplementary material for: Influence of cryoablation versus operation on circulating lymphocyte subsets in patients with early-stage renal cell carcinoma
Source: BMC Cancer. 2024 Jul 10;24:825. doi: 10.1186/s12885-024-12596-w (PMC11238514; doi:10.1186/s12885-024-12596-w)
Supplement: Supplementary file 1 — Additional file 1. [file 12885_2024_12596_MOESM1_ESM.pdf]

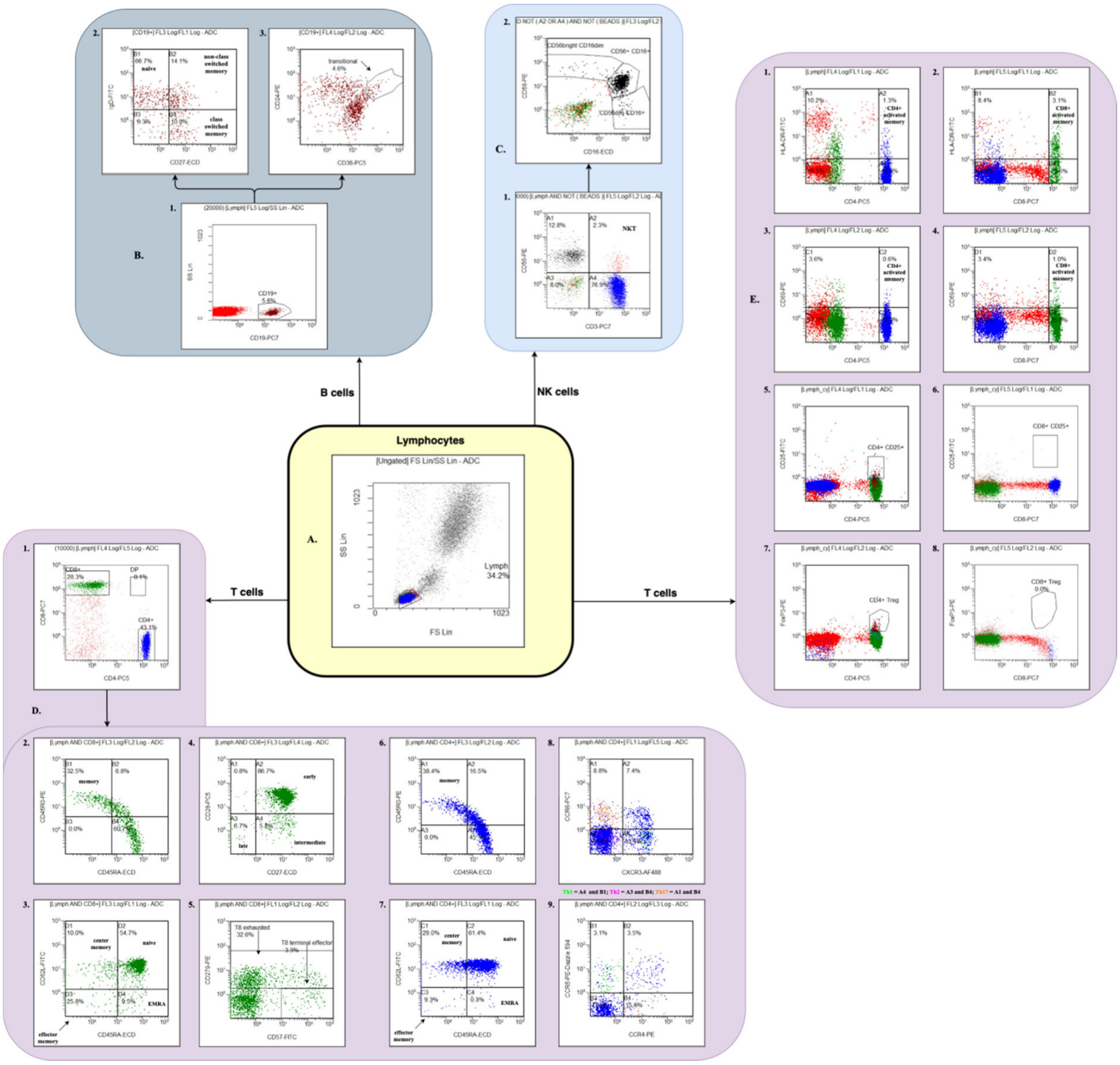

Supplemental Figure 1: **Gating Strategy.** (**A**.) Lymphocytes were identified by using forward scatter (FC) and side scatter (SC). (**B**.) Additional CD19 positivity was used to define B cells (Plot 1), which were further subdivided into naïve (IgD+), class switched memory (CD27+), non-class switched memory (IgD+ CD27+, Plot 2) and transitional B cells (CD24+ CD38+, Plot 3). (**C**.) NKT cells (CD3+ CD56+) were primarily identified (Plot 1). CD3- cells were classified into 3 different subgroups of NK cells (CD56+ CD16+, CD56bright CD56 dim, CD56dim CD16bright, Plot 2).

(D.) T cells were identified by CD4 or CD8 positivity. Both CD4+ as well as CD8+ cells were subdivided into memory (CD45RA- CD45RO+) (Plots 1+6), naïve (CD62L+ CD45RA+), central memory (CD62L+ CD45RA-), effector memory (CD62L- CD45RA-) and effector memory RA+ ("EMRA") (CD62L- CD45RA+) cells (Plots 3+7). To analyze CD8+ cell activity, cells were subdivided into early (CD27+ CD28+), intermediate (CD27+ CD28-), late (CD27- CD28-) (Plot 4), or exhausted (CD279+) and terminal effector (CD279- CD57+) cells (Plot 5). CD4+ T helper cells were classified into Th1 (CXCR3+ CCR4- CCR5+ CCR6-), Th2 (CXCR3- CCR4+ CCR5- CCR6-) and Th17 (CXCR3- CCR4+ CCR5- CCR6+) cells (Plots 8+9). **(E.)** CD4+ and CD8+ cells were also subdivided into activated memory cells (HLA-DR+ or CD69+) (Plots 1-4) and regulatory cells (CD25+ or FoxP3+) (Plots 5-8).

Table S1: Flow Cytometric Analysis of Lymphocytes

B cells	B lymphocytes were identified by the presence of CD19 (CD19-PC7 IM3628) and were further divided into naïve (IgD+ CD27-; IgD-FITC B30652, CD27-ECD B26603), memory (IgD+ CD27+), class switched memory (IgD- CD27+) and transitional (CD24hi CD38hi; CD24-PE IM1428U, CD38-PC5 A07780) subsets.
T cells	Total T lymphocyte counts were obtained using CD3 and Stem-Count as described above. For further T cell analyses, lymphocytes were identified using forward, and side scatter.<br>T lymphocytes were defined by positivity for CD8 or CD4 and were subdivided into naïve (CD62L+ CD45RA+) and memory T cells (CD4+ CD45RA- CD45RO+ /CD8+ CD45RA- CD45RO+), which were further divided into central memory (CD62L+ CD45RA-), effector memory (CD62L- CD45RA), effector memory RA+ (EMRA; CD62L- CD45RA+) and activated CD4+ or CD8+ cells (HLA-DR+ or CD69+) cells, and regulatory cells (CD4+ CD25hi, displaying IL2R+ CD4+ cells).<br>Furthermore, type 1, 2 and 17 CD4+ T helper (Th1/Th2/Th17) cells were identified by using antibodies against CXCR3, CCR4, CCR5 and CCR6. Th1 cells were defined as CD4+ CXCR3+ CCR4- CCR5+ CCR6-, Th2 cells as CD4+ CXCR3- CCR4+ CCR5- CCR6- and Th17 cells as CD4+ CXCR3- CCR4+ CCR5- CCR6+.<br>Within cytotoxic CD8+ T lymphocytes, activated subsets in early (CD28+ CD27+), intermediate (CD28- CD27+) and late (CD28- CD27-) status were identified as well as exhausted (CD279+) and terminal effector (CD279- CD57+) cells. Additionally, CD56+ CD3+ T (NK T) cells were registered.
NK cells	NK lymphocytes were detected as CD56+ cells and subdivided into 3 NK subsets (CD56+ CD16+, CD56dim CD16bright, and CD56bright CD16dim).

Table S2: Fluorochrome-antibody conjugates

Cell population	Antigen	Fluorochrome	Clone	Isotype	Beckman Coulter/Biolegend item number
B	IgD	FITC	IA6-2	IgG2a	B30652
B	CD24	PE	ALB9	IgG1	IM1428U
B, exhausted T	CD27	ECD	1A4CD27	IgG1	B26603
B	CD38	PC5	LS198-4-3	IgG1	A07780
B	CD19	PC7	J3-119	IgG1 kappa	IM3628
Memory T	CD62L	FITC	DREG56	IgG1	IM1231U
Memory T	CD45 R0	PE	UCHL1	IgG2a	A07787
Memory T, Treg	CD45RA	ECD	2H4LDH11LDB9	IgG1	IM2711U
Memory T, activated T, Treg, Th subsets	CD4	PC5	13B8.2	IgG1	A07752
Memory T, Treg, exhausted T, activated T	CD8	PC7	SFCI21Thy2D3	IgG1	737661
Exhausted CD8-T	CD57	FITC	NC1	IgM	B49188
Exhausted CD8-T	CD279	PE	PD1.3	IgG2b	B30634
Exhausted CD8-T	CD28	PC5	CD28.2	IgG1	6607108
Activated T	HLA-DR	FITC	Immu-357	IgG1	IM1638U
Activated T	CD69	PE	TP1.55.3	IgG2b	IM1943U
Treg	CD25	FITC	B1.49.9	IgG2a	IM0478U
Treg	FoxP3	PE	Ab259D	IgG1 kappa	B46031
T/NK	CD56	PE	N901/NK-1	IgG1	A07788
T/NK	CD16	ECD	3G8	IgG1	A33098
T/NK	CD19	PC5	J3-119	IgG1	A07771
T/NK	CD3	PC7	UCHT1	IgG1	737657
Th subsets	CD183 (CXCR3)	AF488	G025H7	IgG1	B68144
	CD194 (CCR4)	PE	L291H4	IgG1	359412
	CD195 (CCR5)	PE/Dazzle 594	J418F1	IgG2b	359126
	CD196 (CCR6)	PC7	B-R35	IgG2a	B68132

FITC fluorescein isothiocyanate, *PE* phycoerythrin, *ECD* phycoerythrin-Texas red, *PC-5* phycoerythrin cyanin, *PC7* phycoerythrin cyanin7, *AF488* Alexa-Fluor 488

Table S3 – Multivariate analysis of lymphocyte subsets regarding age, gender and RCC

	Variables	Coefficients B (95% CI)	p
**Total Lymphocytes**	age (/10 years)	0.936 (0.881-0.994)	0.0310
	gender	0.886 (0.727-1.080)	0.2260
	RCC	0.908 (0.714-1.156)	0.4290
**Total Tcells (CD3+)**	age (/10 years)	0.917 (0.855-0.983)	0.0160
	gender	0.825 (0.656-1.038)	0.0990
	RCC	0.941 (0.712-1.245)	0.6660
**Cytotoxic Tcells (CD8+)**	age (/10 years)	0.878 (0.802-0.961)	0.0060
	gender	1.189 (0.883-1.600)	0.2500
	RCC	0.793 (0.552-1.138)	0.2050
**CD8+ naive**	age (/10 years)	0.558 (0.466-0.667)	0.0000
	gender	0.720 (0.399-1.300)	0.2720
	RCC	2.986 (1.459-6.113)	0.0030
**CD8+ memory**	age (/10 years)	0.868 (0.770-0.978)	0.0210
	gender	1.083 (0.732-1.605)	0.6850
	RCC	0.990 (0.614-1.598)	0.9680
**CD8+ CM**	age (/10 years)	0.758 (0.642-0.894)	0.0010
	gender	0.968 (0.560-1.672)	0.9050
	RCC	2.262 (1.166-4.391)	0.0170
**CD8+ EM**	age (/10 years)	0.887 (0.784-1.004)	0.0570
	gender	1.132 (0.753-1.702)	0.5460
	RCC	0.818 (0.499-1.342)	0.4210
**CD8+ EMRA**	age (/10 years)	1.160 (0.978-1.376)	0.0860
	gender	1.143 (0.650-2.008)	0.6390
	RCC	0.497 (0.251-0.985)	0.0450
**CD8+ early**	age (/10 years)	0.802 (0.731-0.880)	0.0000
	gender	1.089 (0.805-1.475)	0.5750
	RCC	0.972 (0.672-1.405)	0.8770
**CD8+ intermediate**	age (/10 years)	0.941 (0.813-1.089)	0.4070
	gender	0.918 (0.568-1.485)	0.7240
	RCC	0.638 (0.355-1.145)	0.1300
**CD8+ late**	age (/10 years)	0.992 (0.832-1.183)	0.9260
	gender	1.450 (0.813-2.585)	0.2040
	RCC	0.559 (0.277-1.131)	0.1040
**CD8+ exhausted**	age (/10 years)	1.005 (0.863-1.170)	0.9490
	gender	1.219 (0.741-2.006)	0.4310
	RCC	0.436 (0.238-0.800)	0.0080
**CD8+ TE**	age (/10 years)	1.015 (0.830-1.242)	0.8830
	gender	1.405 (0.725-2.723)	0.3090
	RCC	0.624 (0.279-1.398)	0.2480

**CD8+ CD69+**	age (/10 years)<br>gender<br>RCC	0.859 (0.734-1.006)<br>1.633 (0.975-2.738)<br>0.470 (0.251-0.882)	0.0580<br>0.0620<br>0.0190
**CD8+ HLADR**	age (/10 years)<br>gender<br>RCC	0.993 (0.830-1.187)<br>1.114 (0.619-2.005)<br>0.954 (0.467-1.952)	0.9340<br>0.7140<br>0.8970
**CD8+ CD25hi**	age (/10 years)<br>gender<br>RCC	1.256 (0.987-1.598)<br>1.044 (0.473-2.302)<br>0.269 (0.103-0.705)	0.0640<br>0.9140<br>0.0080
**T helper cells (CD4+)**	age (/10 years)<br>gender<br>RCC	0.937 (0.872-1.007)<br>0.755 (0.596-0.956)<br>0.931 (0.698-1.242)	0.0760<br>0.0200<br>0.6230
**CD4+ naive**	age (/10 years)<br>gender<br>RCC	0.865 (0.785-0.953)<br>0.619 (0.449-0.855)<br>0.982 (0.665-1.451)	0.0040<br>0.0040<br>0.9260
**CD4+ memory**	age (/10 years)<br>gender<br>RCC	0.953 (0.884-1.027)<br>0.802 (0.627-1.025)<br>1.018 (0.754-1.374)	0.2000<br>0.0780<br>0.9050
**CD4+ CM**	age (/10 years)<br>gender<br>RCC	0.854 (0.776-0.940)<br>0.680 (0.496-0.934)<br>1.694 (1.154-2.488)	0.0020<br>0.0180<br>0.0080
**CD4+ EM**	age (/10 years)<br>gender<br>RCC	1.056 (0.961-1.161)<br>0.939 (0.687-1.285)<br>0.598 (0.409-0.875)	0.2560<br>0.6910<br>0.0090
**CD4+ EMRA**	age (/10 years)<br>gender<br>RCC	1.467 (1.115-1.931)<br>1.301 (0.525-3.223)<br>0.123 (0.041-0.371)	0.0070<br>0.5650<br>0.0000
**CD4+CD69+**	age (/10 years)<br>gender<br>RCC	0.967 (0.847-1.104)<br>0.596 (0.385-0.922)<br>0.800 (0.470-1.361)	0.6150<br>0.0210<br>0.4050
**CD4+HLADR**	age (/10 years)<br>gender<br>RCC	1.066 (0.968-1.175)<br>0.912 (0.664-1.252)<br>1.094 (0.743-1.610)	0.1900<br>0.5630<br>0.6460
**THC 1**	age (/10 years)<br>gender<br>RCC	1.103 (0.956-1.273)<br>1.049 (0.656-1.676)<br>0.369 (0.208-0.655)	0.1760<br>0.8410<br>0.0010
**THC 2**	age (/10 years)<br>gender<br>RCC	1.025 (0.935-1.125)<br>0.691 (0.510-0.935)<br>1.163 (0.803-1.686)	0.5900<br>0.0180<br>0.4190

**THC 17**	age (/10 years)<br>gender<br>RCC	0.997 (0.912-1.089)<br>0.788 (0.590-1.054)<br>1.032 (0.724-1.472)	0.9400<br>0.1060<br>0.8580
**CD4+ CD25hi**	age (/10 years)<br>gender<br>RCC	0.836 (0.763-0.915)<br>1.036 (0.768-1.398)<br>0.862 (0.598-1.241)	0.0000<br>0.8130<br>0.4190
**CD3+ CD56+**	age (/10 years)<br>gender<br>RCC	1.005 (0.846-1.193)<br>0.669 (0.381-1.176)<br>0.594 (0.299-1.181)	0.9550<br>0.1600<br>0.1350
**Total B cells (CD19+)**	age (/10 years)<br>gender<br>RCC	0.899 (0.813-0.995)<br>0.914 (0.656-1.272)<br>0.654 (0.437-0.978)	0.0390<br>0.5890<br>0.0390
**Naïve B cells**	age (/10 years)<br>gender<br>RCC	0.854 (0.762-0.957)<br>0.870 (0.598-1.266)<br>0.711 (0.450-1.122)	0.0080<br>0.4610<br>0.1410
**Non-class switched memory B cells**	age (/10 years)<br>gender<br>RCC	0.887 (0.774-1.016)<br>0.946 (0.606-1.477)<br>0.697 (0.405-1.199)	0.0820<br>0.8040<br>0.1890
**Class switched memory B cells**	age (/10 years)<br>gender<br>RCC	0.987 (0.867-1.123)<br>0.839 (0.548-1.285)<br>0.563 (0.336-0.946)	0.8360<br>0.4150<br>0.0310
**Transitional B cells**	age (/10 years)<br>gender<br>RCC	0.719 (0.578-0.895)<br>0.661 (0.323-1.351)<br>0.530 (0.222-1.267)	0.0040<br>0.2520<br>0.1510
**Natural killer cells**	age (/10 years)<br>gender<br>RCC	0.914 (0.844-0.990)<br>0.835 (0.642-1.086)<br>1.255 (0.912-1.728)	0.0280<br>0.1750<br>0.1610
**CD56+ CD16+**	age (/10 years)<br>gender<br>RCC	0.905 (0.827-0.991)<br>0.831 (0.617-1.119)<br>1.200 (0.835-1.725)	0.0320<br>0.2190<br>0.3190
**CD56bright CD16dim**	age (/10 years)<br>gender<br>RCC	0.890 (0.823-0.962)<br>0.726 (0.562-0.939)<br>1.112 (0.813-1.520)	0.0040<br>0.0150<br>0.5010
**CD56dim CD16bright**	age (/10 years)<br>gender<br>RCC	0.935 (0.837-1.044)<br>0.955 (0.664-1.374)<br>1.387 (0.891-2.160)	0.2280<br>0.8020<br>0.1450
**CD4/CD8 ratio**	age (/10 years)<br>gender<br>RCC	1.067 (0.996-1.143)<br>0.636 (0.508-0.797)<br>1.174 (0.892-1.544)	0.0640<br>0.0000<br>0.2470
